# Animal study on factors influencing anterograde renal pelvis perfusion manometry

**DOI:** 10.3389/fphys.2024.1258175

**Published:** 2024-11-14

**Authors:** Xin Liu, Xing Li, Limin Liao

**Affiliations:** ^1^ Department of Urology, China Rehabilitation Research Center, Beijing Bo’ai Hospital, Beijing, China; ^2^ Cheeloo College of medicine, Shandong University, Jinan, Shandong, China; ^3^ University of Health and Rehabilitation Sciences, Qingdao, Shandong, China; ^4^ School of Rehabilitation, Capital Medical University, Beijing, China; ^5^ China Rehabilitation Science Institute, Beijing, China

**Keywords:** upper urinary tract urodynamics, partial ureter obstruction, lower urinary tract function, pressure-perfusion study, renal pelvis pressure

## Abstract

**Objects:**

Anterograde renal pelvis perfusion manometry is an effective method to assist in the diagnosis of upper urinary tract obstruction.

**Methods:**

To established a rat model of partial ureteral obstruction to explore the perfusion rate, renal pelvis volume, obstruction sites, contralateral upper urinary tract, and lower urinary tract functions, which may affect anterograde renal pelvis perfusion manometry. To measure the renal pelvis volume using ultrasound. Depending on whether clamped the contralateral ureter and it continuously emptied the bladder, perfused the renal pelvis at rate of 15, 30, 60, 90, or 120 mL/h to measure the pressure synchronously.

**Results:**

The research showed the renal pelvis volume of UPJ and UVJ at 1, 2, 3, and 4 weeks respectively, significantly increased compared with the control group. Comparison of the renal pelvis volume between the UPJ and UVJ groups was not statistically significant. The renal pelvis pressure of UPJ and UVJ was significantly increased compared with the control group, and the UVJ group was greater than the UPJ group. The renal pelvic pressure increased as the perfusion rate increased. Comparing the renal pelvis pressure measured using synchronous bladder emptying with the renal pelvis pressure measured singly, the difference was statistically significant. Comparing the renal pelvis pressure measured using synchronous bladder emptying with measured with a clamped contralateral ureter, the difference was not statistically significant; however, in some groups, the difference was statistically significant. Measuring the renal pelvis pressure singly and clamping the contralateral ureter, the difference was not statistically significant, except in some groups, the difference was significant.

**Conclusion:**

The study suggested that ureter obstruction sites, perfusion rates, renal pelvis volumes, and synchronous bladder emptying affects the renal pelvis pressure. The function of the contralateral upper urinary tract did not affect renal pelvis pressure in the short term.

## 1 Introduction

Upper urinary tract urodynamics is a science that studies the physiologic and pathologic mechanisms of urine production and delivery. The measurement of upper urinary pressure is important to the study of upper urinary tract urodynamics. Upper urinary tract obstruction may lead to abnormal changes in urodynamics. Measuring upper urinary tract pressure can provide a reference and basis for the mechanism, auxiliary diagnosis, treatment strategy, and postoperative evaluation of upper urinary tract diseases.

Upper urinary tract obstruction increases the resistance to urine delivery. Researchers have conducted studies to clarify the critical value of renal pelvis pressure caused by obstruction. In 1973, Whitaker was the first to establish percutaneous pyelocentesis by perfusing the renal pelvis at a constant flow rate of 10 mL/min and expressing the renal pelvis pressure minus the intravesical pressure as the pressure through which a bolus could pass through the ureter. Less than 15cmH_2_O indicated no obstruction, greater than 22cmH_2_O showed obstruction, and 15 to 22cmH_2_O revealed ambiguous obstruction (Whitaker, 1973; Whitaker, 1979). This procedure is suitable for patients whose condition cannot be confirmed through imaging and those with poor renal function accompanied by suspected obstruction; a negative diuretic nephrogram and lumbago pain, suspected interstitial obstruction; a positive diuretic nephrogram concomitant with severe dilatation of the upper urinary tract.

The Whitaker test has been used in clinical practice. Lupton reported on 25 years of experience in a single-center clinical application of the Whitaker test on 145 kidneys suspected of upper urinary tract obstruction, among which 61 patients were confirmed to have obstruction, and 17 had probable abnormal pelvis peristalsis. In patients with idiopathic hydronephrosis, the results were consistent with the diuretic nephrogram in 72% of cases (Lupton and George, 2010). Li and colleagues conducted magnetic resonance urography with the Whitaker test for patients with ileal ureter replacement after surgery. They found that the images and pressure changes of upper urinary tract reconstruction under different perfusion loads were different, aiding in the clinical diagnosis of many suspected cases (Li et al., 2021). Johnston found that the Whitaker test has diagnostic value in patients with suspected the uretopelvic junction (UPJ) or the uretovesical junction (UVJ) obstruction and those with primary ureteral dynamic deficiency (Johnston and Porter, 2014).

The Whitaker test has other clinical value as well. Yang performed the modified Whitaker test combined with image-urodynamics examination for postoperative patients with complex upper urinary tract reconstruction to evaluate the urodynamics and to guide removal of the nephrostomy tube (Yang et al., 2021). Grauer confirmed a diagnosis of a renal ptosis patient with recurrent back pain through the modified Whitaker test, the patient was confirmed to have position-dependent obstruction resulting in elevated renal pelvis pressure (Grauer et al., 2020). All of these studies reflect the significant clinical value of the Whitaker test.

Many studies have shown that ureter obstruction can cause different degrees of kidney damage on the affected side, and after a period of time, the renal function tends to be stabilize with no major changes (Wang et al., 2021; Yuvanc et al., 2021). However, there are few studies about variation in contralateral renal function. Ekinciet reported that obstruction can cause compensatory hyperplasia of the contralateral renal tissue, tubular dilatation, glomerular congestion, and other changes (Ekinci et al., 2018). So far, it is not known whether upper urinary tract urodynamic abnormalities can affect contralateral renal pelvis pressure.

We found that the Whitaker test has several shortcomings, including that the perfusion rate and upper urinary tract volume lead to poor repeatability of experimental results and that false-positive outcomes such as abnormal lower urinary tract dynamics may occur. Due to the unique anatomical characteristics of UPJ and UVJ, the measured renal pelvis pressure cannot fully represent the functional status of the ureter (Burgos et al., 1986). We intend to establish a rat model of partial ureter obstruction and study different renal pelvis volumes and perfusion rates, contralateral ureter function, and the influence of an empty bladder to determine whether these factors can meaningfully affect renal pelvis pressure.

## 2 Materials and methods

### 2.1 Experimental animals

90 SD rats weighing 200–220 g (Beijing SPF Biotechnology Co., Ltd., China). Rats were kept in an environment of 22°C, 50%–60% humidity, good ventilation, a 12-h light/dark cycle, and free access to food and water. All animal procedures were conducted in compliance with the institutional guidelines for the care and use of laboratory animals and were approved by the Animal Care and Use Committee at China Rehabilitation Research Center. All experimental protocols for this study were approved by the Animal Ethics Committee of the China Rehabilitation Research Center (Beijing, China, ID: AEEI-2022-150).

### 2.2 Establish the partial ureter obstruction model

The rats were randomly assigned to an experimental group and a control group, and the experimental group was classified into 1-, 2-, 3-, and 4-week groups with UPJ and UVJ partial obstruction, with 10 rats in each group. The rats were anesthetized using an intraperitoneal injection of 3 mL/kg 3% pentobarbital sodium. The UPJ and UVJ were isolated from the ureter along the pounds central through the retroperitoneum. We placed a 1.5 cm-long 3F ureteral catheter parallel to the ureter and tightened the wire knot when the ureteral wall on both sides of the ureteral catheter was closed. Then we extracted the ureteral catheter, established UPJ and UVJ partial obstruction, administered 5 mg/100 g ceftazidime sodium to prevent infection, and offered clean water after operation. The control group did not undergo operation.

### 2.3 Renal pelvis ultrasonography

The rats were anesthetized using an intraperitoneal injection of 3 mL/kg 3% pentobarbital sodium. We measured the anteroposterior diameter (APD), long diameter (L), and transverse diameter (T) using a Siemens color Doppler ultrasonic diagnostic instrument with a probe frequency of 8 MHz and calculated the renal pelvis volume with the formula V = APD*L*T*π/6 (Janki et al., 2018).

### 2.4 Perfusion-pressure measurement

Before the experiment, rats were restricted from drinking water for 24 h, the rats were anesthetized using an intraperitoneal injection of 3 mL/kg 3% pentobarbital sodium, give a blanket to keep warm, exposing the affected kidney and the contralateral ureter via a retroperitoneal approach. We used a G18/1.3 × 80 mm intravenous puncture needle to puncture the renal pelvis along the hypovascular area at the back of the kidney. Depending on the measurement method, we decided whether to place an indwelling catheter through the urethra to empty the bladder.

**FIGURE 1:**
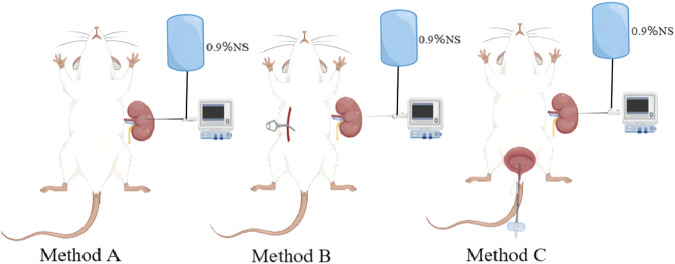
Schematic diagram of different pressure measurement methods.Method **(A)** measure the renal pelvis pressure singly; Method **(B)** clamp the contralateral ureter; Method **(C)** emptied bladder synchronously.

Using an MP150 multi-channel physiological recorder (BIOPAC, United States), we filled the pipes of the manometry and perfusion system with 0.9% normal saline (heat to 38°C), discharged the air, placed the distal end of the tube in the atmosphere, and zeroed at the same horizontal plane as the affected renal pelvis and puncture needle. Subsequently, the pyrheliometer was connected to a three-way tube, and the micro-perfusion pump and pressure sensor were connected. Perfusion was successively performed at 15, 30, 60, 90, and 120 mL/h, and the renal pelvis pressure was measured and recorded according to whether we measured the renal pelvis pressure singly (method A), the contralateral ureter was clamped when the rats began measurements after needle insertion (method B), and the bladder was emptied bladder synchronously (method C). After the adaptive contraction of the renal pelvis, the perfusion measurement was performed, and when the renal pelvis contraction and the curve was stable about 10–15 min, the next cycle of measurement was performed. After recording five datas each time, we took the average value as the final renal pelvis pressure under the corresponding measurement conditions (Figure 1).

### 2.5 Statistical analysis

Data are presented as mean ± standard deviation (‾X ± S). Statistical analysis was performed using SPSS 26 software (IBM, Armonk, NY, United States). An independent sample t-test was used to compare the two groups. Comparison of renal pelvis pressure at different perfusion rates was performed using a paired t-test or One-way analysis of variance. Multiple linear regression analysis and a regression model were used to explore the efficacy of the correlation between parameters and renal pelvis pressure. *P* < 0.05 was considered statistically significant.

## 3 Results

### 3.1 Renal pelvis volume

**FIGURE 2:**
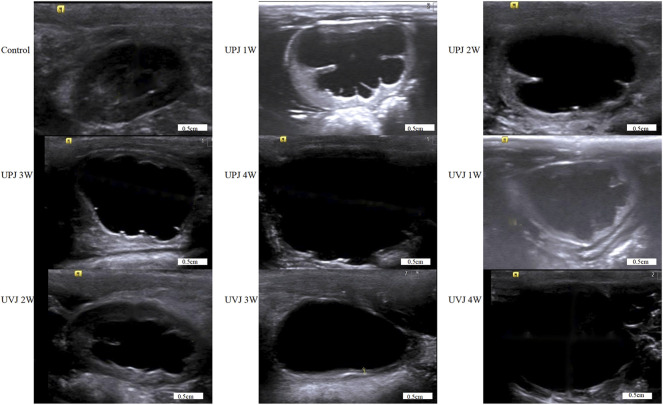
Images of renal pelvis in each group were obtained by B-ultrasound.

The renal pelvis volumes at 1, 2, 3, and 4weeks in the UPJ and UVJ groups compared with the control group, the difference was statistically significant. Comparison of the UPJ and UVJ showed a non-significant difference. (Table 1; Figure 2).

**TABLE 1:** Volume of renal pelvis: cm.^3^.

Control	UPJ 1w	UPJ 2w	UPJ 3w	UPJ 4w	UVJ 1w	UVJ 2w	UVJ 3w	UVJ 4w
0.016 ± 0.007	1.64 ± 0.25^*^	2.43 ± 0.18^*^	2.79 ± 0.17^*^	3.07 ± 0.16^*^	1.65 ± 0.18^*#^	2.28 ± 0.18^*#^	2.57 ± 0.14^*#^	2.98 ± 0.14^*#^

Note: *P < 0.05, ^#^ *P* > 0.05.

### 3.2 Renal pelvis pressure curve

**FIGURE 3:**
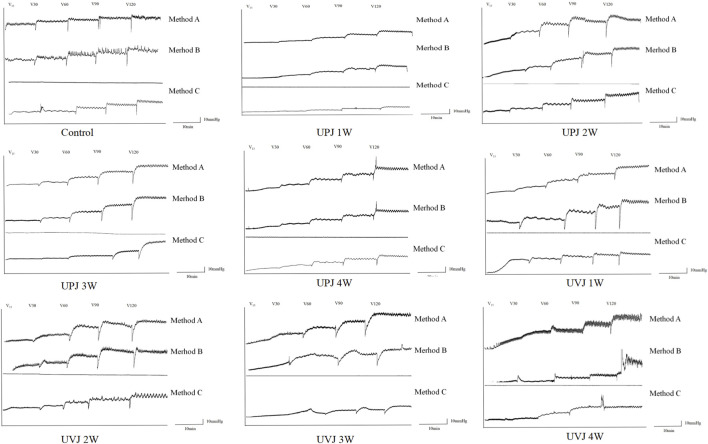
Images of the renal pelvis pressure curve under different perfusion rates, groups, obstruction sites and method.

The images show the Renal pelvis pressure curve under different perfusion rates, groups, obstruction sites and method.(Figure 3).

### 3.3 Renal pelvis pressure at different groups

**FIGURE 4:**
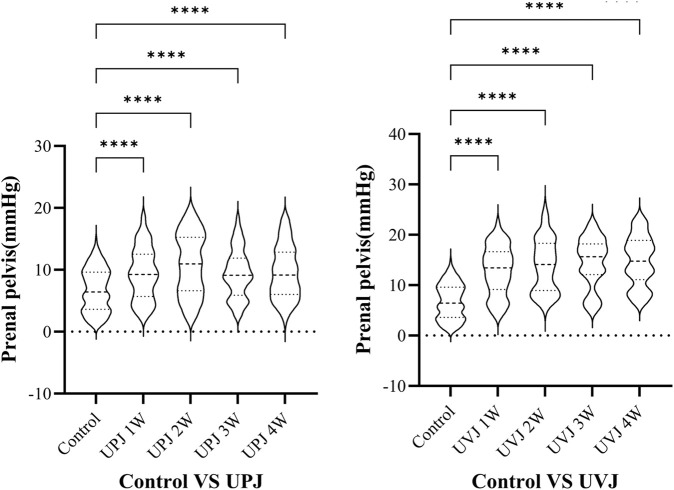
Comparison the pressure between control group and upj or uvj group. Note: ****P < 0.0001.

The renal pelvis pressure in the UPJ and UVJ groups was higher than the control group. (Figure 4).

### 3.4 Renal pelvis pressure at different obstruction sites

**FIGURE 5:**
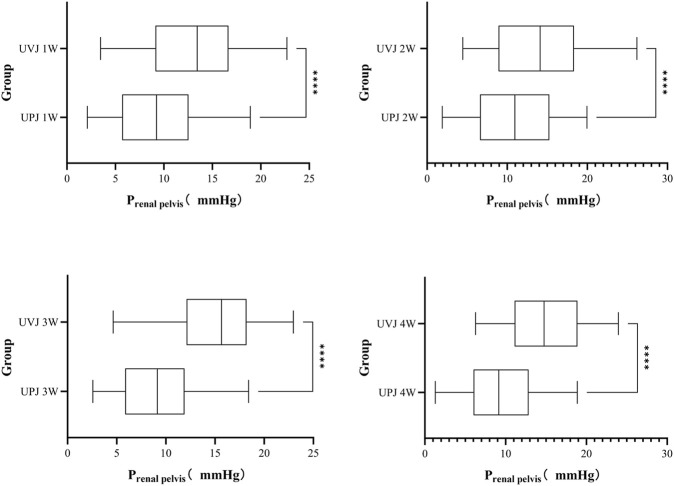
Comparison the pressure between UPJ and UVJ group. Note: ****P < 0.0001.

The renal pelvis pressure in the UVJ group was greater than the corresponding UPJ group. (Figure 5.).

### 3.5 Renal pelvis pressure under different perfusion rates

There were significant differences in renal pelvis pressure under different perfusion rates between the UPJ, UVJ, and control groups. (Table 2).

**TABLE 2:** Comparison the pressure at different perfusion rates: mmHg.

Method_V(mL/h)_	‾x ± s	P	Method_V(mL/h)_	‾x ± s	P	Method_V(mL/h)_	‾x ± s	P
A15-30	−3.49 ± 1.73	<0.001	B15-30	−3.15 ± 1.89	<0.001	C15-30	−3.44 ± 1.89	<0.001
A15-60	−6.38 ± 1.72	<0.001	B15-60	−6.30 ± 2.43	<0.001	C15-60	−6.17 ± 1.86	<0.001
A15-90	−9.10 ± 2.09	<0.001	B15-90	−8.97 ± 2.65	<0.001	C15-90	−8.83 ± 1.96	<0.001
A15-120	−11.60 ± 2.25	<0.001	B15-120	−11.81 ± 2.72	<0.001	C15-120	−11.14 ± 1.93	<0.001
A30-60	−2.89 ± 1.37	<0.001	B30-60	−3.15 ± 1.15	<0.001	C30-60	−2.72 ± 1.47	<0.001
A30-90	−5.61 ± 1.99	<0.001	B30-90	−5.82 ± 1.78	<0.001	C30-90	−5.38 ± 1.91	<0.001
A30-120	−8.11 ± 2.35	<0.001	B30-120	−8.66 ± 2.01	<0.001	C30-120	−7.70 ± 2.00	<0.001
A60-90	−2.72 ± 1.14	<0.001	B60-90	−2.68 ± 1.10	<0.001	C60-90	−2.66 ± 1.12	<0.001
A60-120	−5.22 ± 1.75	<0.001	B60-120	−5.51 ± 1.48	<0.001	C60-120	−4.98 ± 1.37	<0.001
A90-120	−2.50 ± 1.35	<0.001	B90-120	−2.84 ± 1.00	<0.001	C90-120	−2.32 ± 1.00	<0.001

Note: Methods: A: measured the renal pelvis singly; B: clamped the contralateral ureter; C: Emptied the bladder synchronously. V: Perfusion rate (mL/h).

### 3.6 Renal pelvis pressure under different measurement methods

**FIGURE 6:**
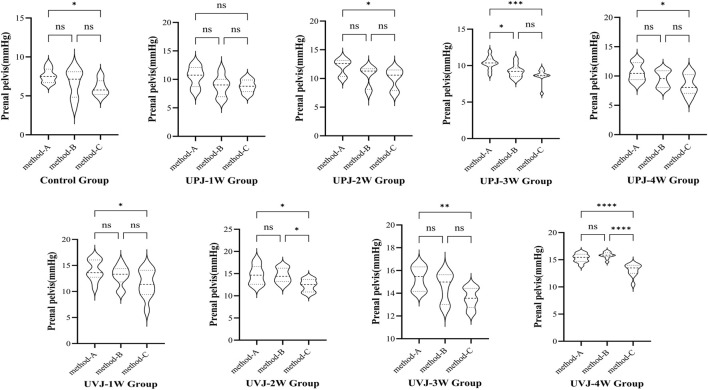
Comparison the pressure between different methods.Note: *P < 0.05; **P < 0.01; ***P < 0.001; ****P < 0.0001.

The renal pelvis pressure measured under the single method was higher than that using the synchronous method, with a statistically significant difference (*P* < 0.05). In most groups between clamped contralateral ureter and single measurement of renal pelvis pressure, the difference was not statistically significant. In some groups, the renal pelvis pressure of clamped contralateral ureter was significantly lower than under the single measurement method. Comparison of the renal pelvis pressure measured with synchronous bladder emptying and clamped contralateral ureter showed no statistical significance, while in some groups, the difference was statistically significant. (Figure 6).

### 3.7 Multiple linear regression analysis

In this study, obstruction time, obstruction sites, renal pelvis volume, manometry method and perfusion rate were used as independent variables, and the renal plevic pressure is used as dependent variables in the multiple linear regression analysis.Multiple linear regression is used to establish the regression equation: the renal pelvis pressure = 0.805 + 0.052*time + 3.854*site + 0.455*renal pelvis volume + 0.104*perfusion rate-1.006*method (F = 1,438.614, *P* < 0.01) and the model constant = 0.805 and the independent variable could explain 91.8% of the renal pelvis pressure. (Table 3).

**TABLE 3:** Multiple Linear Regression Analysis of independent variables vs. the renal pelvis pressure.

Model summary^b^					
Model	R	R^2^	R_adj_^2^	Std.Errors of the estimate	Durbin-Watson
	0.918^a^	0.843	0.842	2.08727	1.007

ANOVA^a^					
Model	Sum of squares	Degree of freedom	Mean square	F	Sig
Regression	31,337.887	5	6,267.577	1,438.614	0.000^b^
Residuals	5,855.376	1,344	4.357		
Total	37,193.263	1,349			

Coefficient^a^									
Model	Unstd.Coefficient		Std.Coefficient	t	Sig	95.0% CI for B		Collinear statistics	
	B	Std.Error	Beta			Lower	Upper	Tolerance	VIF
Constant	0.805	0.200		4.028	0.000	0.413	1.197		
Position	3.854	0.104	0.489	36.989	0.000	3.649	4.058	0.669	1.495
Volume	−0.455	0.165	−0.079	−2.758	0.006	−0.779	−0.131	0.142	4.041
Method	−1.006	0.070	−0.157	−14.462	0.000	−1.143	−0.87	1.000	1.000
Rate	0.104	0.001	0.763	70.479	0.000	0.101	0.107	1.000	1.000
Time	0.052	0.015	0.091	3.428	0.001	0.022	0.082	0.166	3.006

^a^ Predictive variable: (constant), Time, Rate, Method, Position, Volume.

^b^ Dependent variable: Pressure.

## 4 Discussion

Different noninvasive imaging examinations can be used to diagnose most abnormal upper urinary tract urodynamics. However, the diagnostic value of upper urinary tract manometry for urodynamic abnormalities cannot be ignored. Whitaker proposed pyelostomy and perfused the renal pelvis at a constant rate of 10 mL/min while recording the pressure in the renal pelvis and bladder to aid the diagnosis of upper urinary tract obstruction. However, many subsequent studies have found that patients with chronic alcohol consumption and interstitial obstruction may have false-negative results when perfused at a rate of 10 mL/min (Farrugia and Whitaker, 2019).

When Djurhuus and colleagues performed the Whitaker test on 28 patients with upper urinary tract obstruction, 9 patients with severe hydronephrosis needed a perfusion rate higher than 10 mL/min to obtain correct obstructive renal pelvis pressure, indicating that different degrees of hydronephrosis may affect the pressure (Djurhuus et al., 1985). Some studies have established acute and chronic ureteral obstruction models and included perfusion rate experiments at 1, 5, and 10 mL/min, finding that perfusion rate and renal pelvis volume may be the key factors affecting the reliability of Whitaker test results (Ryan et al., 1989).

In our study, the renal pelvis volumes at 1, 2, 3, and 4 weeks in the UPJ and UVJ groups increased significantly. There was no significant difference between the UPJ and UVJ groups renal pelvis volume. The renal pelvis pressure in the UPJ and UVJ groups was significantly higher than in the control group. The renal pelvis pressure in the UVJ group was higher than the UPJ group. The renal pelvis pressure increased gradually with renal pelvis volume, we speculate that the reason is that the pressure increases with volume during maintenance of upper urinary tract compliance balance. Although the comparison of renal pelvis volume between the UPJ and UVJ groups was not statistically different, actual measurements showed that the pressure in the UVJ group was higher than the UPJ group. Partial ureter obstruction at the UVJ may have led to dilation of the whole ureter, which may have increased the volume of the upper urinary tract and, thus, renal pelvis pressure.

In other studies, the renal pelvis pressure peaked at 2w after ureteral ligation, then gradually decreased and remained constant at 8w (Wen et al., 1998). Koff studied an animal model of chronic ureter partial obstruction, finding that renal pelvis compliance allowed it to adapt to increasing capacity under low pressure (Koff, 2019). As the pelvis volume increases, the pressure increases to a critical point, after which a slight change in the volume of the pelvis causes a significantly increase in pressure. As the renal pelvis volume increases significantly, hydronephrosis increases, the pressure decreases gradually, and the pressure-volume of the partially obstructed renal pelvis reaches equilibrium.

The decrease in renal pelvis pressure is caused by a decrease in glomerular filtration rate and regurgitation of renal pelvis urine to the renal tubules, veins, lymphatics, and renal interstitium (Strobel et al., 2016). In addition, dilation compliance is different across renal pelvis types. The renal parenchyma restricts the intrarenal pelvis and is difficult to dilate, while the extrarenal pelvis is easy to dilate outwardly, and its pressure drops rapidly (Chavez-Iniguez et al., 2020). Our experiment was conducted on rats whose renal pelvis was mainly intrarenal, and the study endpoint was 4w, which was shorter than that of the previously studies. Most of the renal pelvis volume was still in the acute ascending stage; thus, the renal pelvis pressure increased with increasing renal pelvis volume.

The study found that regardless of whether the control or UPJ or UVJ groups were at different perfusion rates, the renal pelvis pressure was significantly different. With an increased perfusion rate, the pressure increased. The flow of perfusion in the upper urinary tract conforms to the hydrodynamics of the Poiseuille formula (Zheng et al., 2021). Therefore, the renal pelvis pressure also increases with the perfusion rate. Hopf conducted the Whitaker test on patients after pyeloplasty and found that the perfusion rate of 10 mL/min can cause a false increase in renal pelvis pressure in some patients (Hopf et al., 2016). Therefore, perfusion rate in the study was questioned, but they did not conduct further study. Lupton measured renal pelvis pressure in patients with upper urinary tract dilatation after useing diuretic and found almost no significant changes in the renal pelvis pressure at a perfusion rate of 10 mL/min. The renal pelvis pressure gradually increased to a stable level when the perfusion rate was increased to 30 mL/min (Lupton et al., 1985). These studies are consistent with the experimental phenomena that we have observed. However, one study measured the renal pressure at the upper, middle, and lower renal calyces and outlet pelvis after 100–150 mL/min perfusion with pig kidneys and found no statistical difference when monitoring pressure among groups under different perfusion rates (Zhu et al., 2016). Jens Mortensen found a renal resting pressure of 0.3–14.7cmH_2_O through perfusion of 40 healthy pig kidneys. The renal pelvis pressure difference was only 3.7cmH_2_O after perfusion of 8–20 mL/min, with a slight fluctuation range (Mortensen et al., 1982; Mortensen et al., 1983). Because upper urinary compliance is inversely proportional to renal pelvis pressure, false positives may occur when the perfusion rate is too fast or when the patient uses diuretics and drinks excessive water.

In contrast, an inadequate perfusion rate may cause some cases of mild obstruction to be missed. Therefore, when clinically performing the pressure-perfusion test, the perfusion rate should be increased, and the pressure value of the renal pelvis with obstruction should be reduced for severely dilated patients. The pressure value of the renal pelvis with obstruction should be decreased for patients with mild dilated or retroperitoneal adhesion.

Whitaker also suggested that renal pelvis pressure measured by perfusion pressure measurement was the absolute pressure of the whole measuring system. After zero adjustments of the whole system, the intra-abdominal pressure should be subtracted (taking intravesical pressure as the intra-abdominal pressure), and the absolute renal pelvis pressure minus the intravesical pressure is equal to the relative pressure, which is expressed as the pressure required by the bolus passing through the obstruction site (Whitaker, 1973). Therefore, whether intravesical pressure should be measured synchronously at different obstruction sites is still undetermined. The research established obstruction models for specific sites in the UPJ and UVJ and found that the renal pelvis pressure measured by synchronous bladder emptying was significantly lower than that measured singly. This phenomenon is consistent with what Whitaker described in the experiment. Jones conducted the Whitaker test on patients with upper urinary tract obstruction and poor bladder compliance, finding that the degree of bladder filling significantly affected upper urinary tract urodynamics. Subsequently, he conducted a study on patients with normal bladder compliance, finding that the rate and degree of bladder filling also affected upper urinary tract urodynamics (Jones et al., 1988).

Other studies have found that the influence of intravesical pressure and detrusor pressure on upper urinary tract urodynamics can be predicted by measuring intravesical pressure and detrusor pressure during the early, middle, and late bladder-filling periods and that the intravesical pressure and detrusor pressure in the middle and late periods were more sensitive and specific in predicting the urodynamic changes in the upper urinary tract (Lyu et al., 2022). This may be because, under normal circumstances, urine is transported to the bladder as a bolus, and continuous perfusion breaks this physiologic mode of transport. After the perfusion liquid fills the bladder, the antireflux mechanism at the vesicoureteral disappears, resulting in increased pressure in the upper urinary tract. The effect of bladder filling on upper urinary tract urodynamics may be altered if the bladder is emptied continuously and synchronously with an indwelling catheter while the renal pelvis pressure is measured.

Mayo found that when the obstruction was located in the UPJ or upper ureter, simultaneous intravesical pressure measurement may not be necessary after the renal pelvis and bladder are emptied before manometry and after standard and strict *in vitro* zeroing. No significant change in renal pelvis pressure was observed on normal ureter manometry, even when the upper urinary tract was dilated due to bladder filling. However, in cases of suspected UVJ obstruction, renal pelvis hypertension with bladder filling was found (Mayo, 1983). Based on these studies, we recommend that the renal pelvis and bladder be emptied entirely before manometry and standardized using *in vitro* zeroing. Bladder emptying should be synchronized to reduce the influence of atmospheric and intraperitoneal pressure and bladder filling on upper urinary tract urodynamics.

The study also found no statistically significant difference in renal pelvis pressure between the clamped contralateral ureter and the single measurement of renal pelvis pressure in most groups. But in some groups, the clamped contralateral ureter-measured renal pelvis pressure was significantly lower than the single measurement method. The measurement error may have been caused by incomplete air emptying, poor sealing, or other reasons in the pressure measuring system during the experiment. However, the overall trend of experimental results is significant, and such measurement errors are acceptable. Therefore, whether abnormal contralateral upper urinary tract urodynamics have long-term effects on the pther side needs further study.

There are also limitations to our study. Firstly, a partial ureteral obstruction model was established, but due to the limitations of detection techniques and evaluation criteria, the degree of obstruction was not measured. No studies of patients were conducted due to ethical constraints. Our prediction model involves a lot of independent variables.These defects may have led to missing reference values in the clinical research methods and conclusions. However, we found that the obstruction site, perfusion rate, renal pelvis volume, and other factors affected the renal pelvis pressure, suggesting that individual diagnosis should be carried out in the clinical setting.

## 5 Conclusion

The research suggested that the obstruction site, perfusion rate, renal pelvis volume, and whether the bladder was emptied synchronously during measurement may affect the renal pelvis pressure, while the function of opposite upper urinary tract does not affect the other side renal pelvis pressure in the short term.Our regression model has a good precision and provides a reference for evaluating the renal pelvis pressure. However, our prediction model still needs multi-center and randomized controlled validation. In order to obtain a simple and convenient non-invasive model for predicting renal pelvis pressure.

## Data Availability

The original contributions presented in the study are included in the article/supplementary material, further inquiries can be directed to the corresponding author.
